# West Africa - A Safe Haven for Frogs? A Sub-Continental Assessment of the Chytrid Fungus (*Batrachochytrium dendrobatidis*)

**DOI:** 10.1371/journal.pone.0056236

**Published:** 2013-02-13

**Authors:** Johannes Penner, Gilbert B. Adum, Matthew T. McElroy, Thomas Doherty-Bone, Mareike Hirschfeld, Laura Sandberger, Ché Weldon, Andrew A. Cunningham, Torsten Ohst, Emma Wombwell, Daniel M. Portik, Duncan Reid, Annika Hillers, Caleb Ofori-Boateng, William Oduro, Jörg Plötner, Annemarie Ohler, Adam D. Leaché, Mark-Oliver Rödel

**Affiliations:** 1 Museum für Naturkunde, Leibniz Institute for Research on Evolution and Biodiversity, Berlin, Germany; 2 Department of Wildlife and Range Management, Faculty of Renewable Natural Resources, CANR, KNUST, Kumasi, Ghana; 3 Department of Biology and Burke Museum, University of Washington, Seattle, Washington, United States of America; 4 Department of Life Sciences, Natural History Museum, London, United Kingdom; 5 Institute of Zoology, London, United Kingdom; 6 Unit for Environmental Research, North-West University, Potchefstroom, South Africa; 7 Charité - University Medicine Berlin, Institute of Microbiology and Hygiene, Berlin, Germany; 8 Museum of Vertebrate Zoology and Department of Integrative Biology, University of California, Berkeley, California, United States of America; 9 Royal Society for the Protection of Birds, Across the River - A Transboundary Peace Park for Sierra Leone and Liberia, Kenema, Sierra Leone; 10 Forestry Research Institute of Ghana, Fumesua, Kumasi, Ghana; 11 Muséum National d'Histoire Naturelle, Département Systématique et Evolution, Origine, Structure et Evolution de la Biodiversité, Paris, France; Imperial College Faculty of Medicine, United Kingdom

## Abstract

A putative driver of global amphibian decline is the panzootic chytrid fungus *Batrachochytrium dendrobatidis* (*Bd*). While *Bd* has been documented across continental Africa, its distribution in West Africa remains ambiguous. We tested 793 West African amphibians (one caecilian and 61 anuran species) for the presence of *Bd*. The samples originated from seven West African countries - Bénin, Burkina Faso, Côte d'Ivoire, Ghana, Guinea, Liberia, Sierra Leone - and were collected from a variety of habitats, ranging from lowland rainforests to montane forests, montane grasslands to humid and dry lowland savannahs. The species investigated comprised various life-history strategies, but we focused particularly on aquatic and riparian species. We used diagnostic PCR to screen 656 specimen swabs and histology to analyse 137 specimen toe tips. All samples tested negative for *Bd*, including a widespread habitat generalist *Hoplobatrachus occipitalis* which is intensively traded on the West African food market and thus could be a potential dispersal agent for *Bd*. Continental fine-grained (30 arc seconds) environmental niche models suggest that *Bd* should have a broad distribution across West Africa that includes most of the regions and habitats that we surveyed. The surprising apparent absence of *Bd* in West Africa indicates that the Dahomey Gap may have acted as a natural barrier. Herein we highlight the importance of this *Bd*-free region of the African continent - especially for the long-term conservation of several threatened species depending on fast flowing forest streams (*Conraua alleni* (“Vulnerable”) and *Petropedetes natator* (“Near Threatened”)) as well as the “Critically Endangered” viviparous toad endemic to the montane grasslands of Mount Nimba (*Nimbaphrynoides occidentalis*).

## Introduction

Amphibian populations are declining in many regions of the world [1]. This is due to a number of causes. Besides the main contributors, like destruction, alteration and fragmentation of habitats, an often suggested cause is a fungal pathogen of the order Chytridiales (*Batrachochytrium dendrobatidis* Longcore *et al*., 1999 - hereafter referred to as *Bd*) which induces the disease chytridiomycosis. The link between declining populations and *Bd* has been subject to a number of reviews [2], [3], [4], [5], [6], [7], [8]. So far it has been responsible for declines in Australia [9], [10], [11], New Zealand [12], Central America [13], [14], [15], [16], [17], [18], North America [19], [20], [21] and Europe [22]. *Bd* has also been detected in many other regions (see [23] for the most recent worldwide compilation), but not associated with declines.

**Figure 1:**
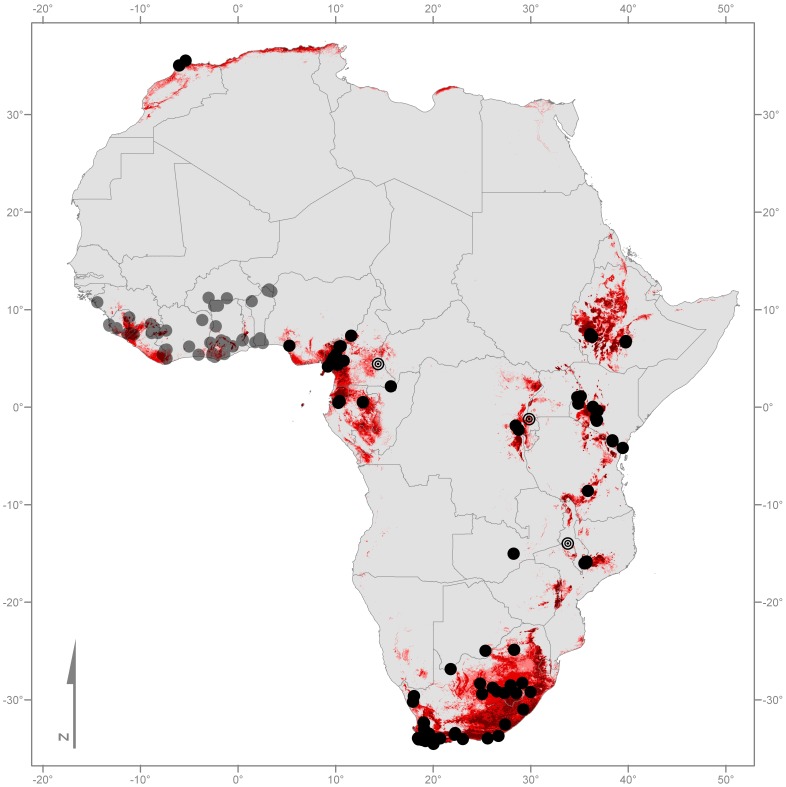
Map of confirmed records of *Bd* on the African continent (black dots). Grey transparent dots represent the West African localities with negative *Bd* records. The hollow black circles indicate *Bd* positive localities [87] which were not used for modelling. The three red colours represent the geographical extent of three different models, predicting the potential distribution of *Bd*. Modelling is based on the conditions of sites with confirmed presence of the pathogen (light red = maximum; red = mean; dark red = minimum; for niche parameters see Table 2).

Currently African records are widespread in southern and eastern Africa, including eastern parts of the Democratic Republic of Congo. These are complemented by very recent additions from Nigeria [24], [25], [26], Cameroon [27], [28] and Gabon [29] (Fig. 1). So far no information has been reported about the pathogen's presence in West Africa. In addition to investigating the pathogen's presence with molecular or histological methods, we infer the likelihood of *Bd* occurrences using environmental niche modelling (ENM). ENM models the Grinnellian niche measured by scenopoetic variables [sensu 30]. This tool has been shown to contribute significantly to our understanding of current species distributions [e.g. 30], [31], [32] and has already been used to model the distribution of pathogens, including that of *Bd* [e.g. 33], [34], [35], [36], [37], [38], [39], [40]. Potential distributions predicted by the models may then guide future surveys aimed at detecting the focal organism [e.g. 41], [42] and preventive measures.

Herein, we compare extensive field surveys for *Bd* based on samples from seven West African countries with results of detailed African continental ENMs, which include the most recent *Bd* positive records. Our findings are discussed with a special focus on common species and on species which are potentially highly threatened by the fungus because of their high niche overlap with *Bd*.

## Materials and Methods

### Ethics Statement

All work complies with the guidelines for the use of live amphibians and reptiles in field research compiled by the American Society of Ichthyologists and Herpetologists (ASIH), The Herpetologists' League (HL) and the Society for the Study of Amphibians and Reptiles (SSAR). For ethical issues concerning the toe clips we refer to [43], we followed recommendations therein.

Permits were issued by the respective companies, institutions, ministries as well as government bodies: Bénin - Faculté des Sciences Agronomiques, Département d'Aménagement et de Gestion de l'Environnement, Laboratoire d'Ecologie Appliquée, Université d'Abomey-Calavi on behalf of the Centre National de Gestion des Réserves de Faune and the Ministère de l'Environnement et de la Protection de la Nature; Burkina Faso - Unité de Formation et de Recherche en Sciences de la Vie et de la Terre, Département de Biologie et Physiologie Végétales, Laboratoire de Biologie et Ecologie Végétales, Université de Ouagadougou on behalf of the National Centre of Scientific and Technology Research of Burkina Faso; Côte d'Ivoire - Ministère de l'Environnement et du Cadre de Vie, Direction de la Protection de la Nature; Ministre de l'Enseignement Supérieur et de la Recherche Scientifique, Direction de la Recherche; Ministère de la Construction et de l'Environnement, Direction de la Protection de la Nature; Ministère de l'environnement et de la Forêt, Direction de la Protection de la Nature; Société de Développement des Forêts; Ghana - Wildlife Commission of the Forestry Commission of Ghana; Guinea - Ministère de l'Agriculture, de l'Elevage, de l'Environnement et des Eaux et Forêts; Ministère de l'Education Nationale et de la Recherche Scientifique, Direction Nationale de la Recherche Scientifique et Technologique; Centre de Gestion de l'Environnement du Nimba-Simandou; Projet des Nations Unies de Développement; Comités Villageois de Surveillance; Ministère du Développement Durable et de l'Environnement, Direction Nationale des Forets et Faune; Société des Mines de Fer de Guinée; Liberia - Forestry Development Authority, Office of the DMD/Forest Conservation; Arcelor-Mittal; Sierra Leone - Ministry Agriculture, Forestry and Food Security, Forests Conservation and Wildlife Unit, Wildlife Conservation Forestry Division.

The “Bundesamt für Naturschutz”, Bonn issued CITES import permits (*Nimbaphrynoides o. occidentalis*; E-3117/07 and E-4074/08; *Nimbaphrynoides o. liberiensis*; E-4509/07), the “Le Directeur Nationale de la Protection de la Nature” (2007/00314) and “L'organe de Gestion CITES Guinée” (2008/0049) in Guinea (*Nimbaphrynoides o. occidentalis*) and the “Forestry Development Authority” in Liberia (01, *Nimbaphrynoides o. liberiensis*) the respective CITES export permits.

### Sampling techniques

**Figure 2:**
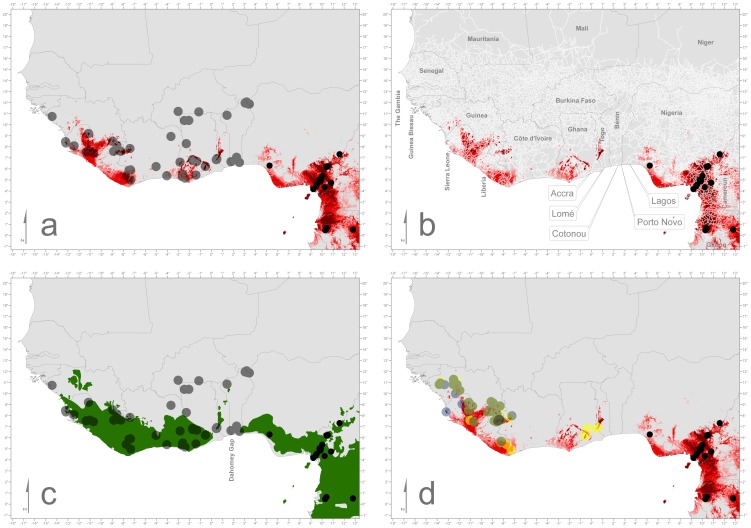
Detailed maps of West Africa. From top to bottom, depicting the most western positive records of *Bd* (black) and the negative records (transparent grey) (2a). Figure 2b indicates in white transparent lines the transport system (roads) of the region. If *Bd* is transported via humans, the area around Accra (Ghana) is most likely to be the point of introduction (well connected via transportation routes and highly suitable environment). Further shown (2c) are the extents of the potentially forest regions (green) with the Upper Guinea Forests west of the Dahomey Gap [after 131], [2a]. In 2d the known point localities of *Conraua alleni* (transparent yellow), *Petropedetes natator* (transparent blue) (light green = overlapping localities), and *Nimbaphrynoides occidentalis* (dark green) are depicted.

Anurans were detected via visual, acoustic or opportunistic searches during the rainy seasons 1993, 1995, 2001 to 2005 and 2009 to 2011. Terrestrial and arboreal species were captured by hand and aquatic species, notably from the family Pipidae, by net. Digging was performed to sample fossorial species such as caecilians. Overall we screened 793 amphibians from 62 species (see Appendix S1 & S2) which originated from 64 sites throughout the region (Table 1) as well as live individuals destined for export at Accra airport, Ghana (Figs. 1, 2a & b).

**Table 1:** Number of amphibian samples per West African country tested for the presence of *Bd*.

Country	Swab	Toe/Skin	Total
Bénin	120	13	133
Burkina Faso	0	3	3
Côte d'Ivoire	29	26	55
Ghana	254	36	290
Guinea	243	44	287
Liberia	10	4	14
Sierra Leone	0	11	11
**Total**	656	137	793

swab = molecular investigation of swab sample; toe/skin = histological examination of toe tips (anurans) and skin pieces (caecilians).

We used two methods: (i) epithelial swabbing and (ii) histology of phalanges to sample for *Bd* [44], [45]. Cotton swabs were utilised to brush the *Bd* sensitive areas of each individual live frog including the ventral surface of each thigh, hind foot and pelvis. Swabs were either placed in 95% ethanol or sprayed with ethanol and stored dry or stored dry directly and kept away from heat [46]. Toe clips were obtained from preserved adult frogs. A piece of dorsal skin was cut one third from the anterior tip of the body and stored in ethanol from preserved caecilians. Toe and skin samples were fixed and stored in 95% ethanol. The samples were analysed at the Museum für Naturkunde, Berlin (MfN; 78 swabs), the North-West University, Potchefstroom (NWU; 105 swabs; 137 toe tips for histology), the University of Washington (UW; 103 swabs) and the Institute of Zoology, London (IoZ; 372 swabs).

Voucher specimens and preserved individuals were euthanized (using either MS-222 or chlorobutanol), preserved in 75% ethanol and are deposited at MfN (134 specimens) or the Burke Museum of Natural History and Culture at UW (103 specimens).

### Laboratory techniques

DNA was extracted with DNeasy extraction kits (Qiagen) following manufacturers protocol. DNA extractions were stored at −80°C (MfN, NWU, IoZ) or 4°C (UW) prior to analysis. At all laboratories, standards of known zoospore concentrations (100, 10, 1 and 0.1 zoospore genomic equivalents (IoZ, NWU, UW)) or ITS copies (169 copies per zoospore (MfN)) and a negative control were used in each diagnostic assay.

At MfN, NWU and IoZ, DNA was analysed using *Bd*-specific primers (ITS-1/5.8-S) and following the RT-qPCR protocol of Boyle *et al*. [47]. At IoZ, bovine serum albumin (BSA) was included in the Taqman mastermix to minimise inhibition of the PCR [48]. The PCR profile was: 5 min at 96°C followed by 50 cycles of 10 s at 96°C and 1 min at 60°C. At all laboratories, a positive result consisted of a clearly sigmoid curve in duplicate samples.

At UW, DNA was analysed by conventional PCR [49] and visualised on a 1.5% agarose gel. To verify that DNA extractions were successful, frog 16 s rRNA (*16S*) was amplified for each sample using standard amphibian primers [50]. As an additional positive control, the universal fungus primers *ITS-4/ITS-5* [51] were used to amplify DNA from various (non-*Bd*) chytrid genera that were extracted from epithelial swabs. The presence/absence of *Bd* was tested by using the *Bd*-specific primers *Bd1a/Bd2a* [49].

Toe clips were dehydrated in an alcohol series (70%, 96% and 2×100% alcohol), elucidated with xylene and infiltrated with paraffin wax at 60°C. Following the wax infiltration the tissues were embedded in paraffin wax blocks using a SLEE MPS/P2 embedding centre and sectioned at 6 µm with a Reickert-Jung 2050 automated microtome. Sections were stained with Mayer's haematoxylin and counter stained with eosin. Slides were then examined under a Nikon Eclipse E800 compound microscope for the presence of *Bd* using the criteria described in Berger *et al*. [52], [53].

### Environmental Niche Modelling

ENM is a statistical modelling tool where a priori set algorithm searches relationships within the data (as opposed to process based modelling). Our ENM relies on maximum enthropy principles (using the software Maxent 3.3.3.k [54], [55], [56]). The approach basically compares the values of the variables at the sites where a species is present against a background sampled from sites with no presences. Maxent uses machine learning to maximise the entropy function; but see Elith *et al*. [32] for a detailed description of the statistics. Despite the number of available algorithms, Maxent is one of the best ENM techniques when using presence-only data [e.g. 57], [58].

Herein we report absence of *Bd*. Nevertheless, the true absence of organisms is in general difficult to ascertain (e.g. compare the findings from [59] and [29]). Therefore we applied the most conservative method using only confirmed presences from the African continent with a high spatial certainty for our ENMs (n = 112 reported records; see Appendix S3). The aim was to model the likely geographic distribution of *Bd* and strictly avoid type II errors.

We used 17 environmental parameters on a 30 arc second grid (which equals roughly 1 km^2^) for the whole African continent as variables in our ENM. All parameters were continuous (not categorical) and are classified into three broad categories: climate, environment and altitude. The climate variables comprised ten parameters, all averaged from 1950 to 2000. Five environmental parameters were obtained from two satellite imagery data sets with different spectral sensitivities (SPOT4 & MODIS). Altitude was converted into two parameters calculated from a radar derived data set (SRTM) (see Table 2).

**Table 2:** Environmental parameters used in the environmental niche modelling (ENM) approach with a short description of the parameter and the source of the original data.

No.	Category	Parameter	Description	Original source
1	climate	tmax_low	lowest value of the maximum temperatures	[132]
2	climate	tmax_high	highest value of the maximum temperatures	[132]
3	climate	tmax_std	standard deviation the maximum temperatures	[132]
4	climate	tmin_low	lowest value of the minimum temperatures	[132]
5	climate	tmin_high	highest value of the minimum temperatures	[132]
6	climate	tmin_std	standard deviation of the minimum temperatures	[132]
7	climate	prec_high	highest precipitation value (wettest month)	[132]
8	climate	prec_low	lowest precipitation value (driest month)	[132]
9	climate	prec_std	standard deviation of the precipitation	[132]
10	climate	prec_sum	total annual precipitation	[132]
11	environment	glc2	vegetation derived from the near-infrared (0.78–0.89 µm) wavelength of the SPOT4 satellite	[133]
12	environment	glc3	vegetation derived from the red (0.61–0.68 µm) wavelength of the SPOT4 satellite	[133]
13	environment	bare	percentage of bare ground (MODIS)	[134]
14	environment	herb	percentage of herbaceous ground cover (MODIS)	[135]
15	environment	tree	percentage of woody vegetation (MODIS)	[136]
16	altitude	srtm_c	elevational contrast calculated from the SRTM30 dataset using a 3×3 moving window	[137]
17	altitude	srtm_v	elevational variance calculated from the SRTM30 dataset using a 9×9 moving window	[137]

Parameters 1–10, calculated in two steps: i) an average (from the years 1950 to 2000) for each month (January to February), thus leading to 12 averages; (ii) calculation as detailed in the main text. Parameters 11–12: calculated from the annual average of the year 2000. Parameters 13–15: extracted from the 500 m MODIS vegetation continuous fields dataset, which are derived from monthly composites that are in turn derived from eight day composites. All 7 bands were used and smoothed via a 4×4 rectangular neighbourhood function. Parameters 16–17: after calculation data were ln-transformed and multiplied by 10 to assure compatibility with other environmental parameters.

In total we calculated 100 ENMs. Models were replicated using sub sampling. For each model, points were randomly allocated into two groups: 70% (n = 79) for model training and 30% (n = 33) for model testing. From these 100 models three average models were derived: maximum, mean and minimum predictions gained. The maximum, mean and minimum models used the average 10 percentile thresholds over all 100 models to gain three binomial models. Models were validated via the area under the curve (AUC) criterion, which refers to the receiver operating characteristic (ROC) curve. This measurement is threshold-independent and commonly used for such models (e.g. [57]).

## Results and Discussion

Despite our extensive sampling on a species and geographical level, we did not detect any evidence of *Bd* in the investigated sites, neither by molecular (at least, any strain known to cause severe chytridiomycosis [60]) nor by histological investigations. Hence, the only region in sub-Saharan Africa without any confirmed records remains the Upper Guinea Forests and the surrounding savannahs.

One positive *Bd* record from Ghana [61] is often cited in the literature and has been used for ENMs [33], [34], [37]. However, it was excluded from our ENM analysis because the specimen stems from the pet trade, has an unknown origin and was tested after being imported into the US. Thus, the specimen could have contracted the pathogen from anyone of a number of possible sources within the trade pathway. Further support for our decision stems from finding that infections at the population level are highly dependent on the density of individuals [62], [63]. Crammed conditions are common in the pet trade and prevalence is high in traded amphibians [64], [65], [66], [67]. In addition, no other *Bd* record was reported from Ghana (n = 292, this paper).

### Continental Modelling

In contrast to these findings our ENMs show that *Bd* could potentially occur in West Africa. So far *Bd* has never been recorded west of Okomu National Park, which lies east of Lagos, Nigeria (see Figs. 1 & 2b). As the fungus prefers moist and comparatively cooler environments [see 68], [69], [70], [71], [72], [73], [74], [75], [76], we hypothesise that the Dahomey Gap, a naturally non-forested stretch ranging from eastern Ghana to western Nigeria, consists of unsuitable habitats and therefore provides a distributional barrier (Fig. 2c). However, this hypothesis must be treated cautiously because *Bd* can survive outside its preferred temperature range [69], [77] and could therefore cross the Dahomey Gap. In addition a number of other factors may influence its persistence as well (e.g. life-history stage at exposure [78], [79], host immunity [80], host stress levels [81] and anthropogenic influences [82], [83]).

Overall the ENMs performed well, with a mean training AUC of 0.979±0.002 and testing AUC = 0.967±0.010. All 17 selected variables contributed to the models. The highest contribution came from the “minimum precipitation” (prec_low 35.3%), followed by the “variance in elevation” (srtm_v 22.6%) and the “lowest value of the maximum temperatures” (tmax_low 17.5%). Jackknife testing revealed “highest value of the maximum temperatures” (tmax_high) as the variable with the greatest information content when used alone (for details see Appendix S4).

Until now no fine-grained continental ENM existed, only coarser ones (2.5 arc minutes) on a global scale [see 37], [40]. Our models showed that *Bd* could occur in the investigated region but not as widespread as in some other parts of Africa. Only a few West African areas were predicted as suitable for *Bd*. These are primarily the comparatively wetter or higher altitude areas of the Upper Guinea forests (see Fig. 2a). Our modelling results show a picture different to the recent global modelling approaches for *Bd* [33], [35]. The main differences are that large areas in Angola, Namibia and Zambia predicted by Ron [33] and Rödder *et al*. [35] are not predicted in our approach. Other differences concern areas in western Africa where our ENM predict a smaller range compared to Ron [33] and Rödder *et al*. [35]. Interestingly large areas in Ethiopia are predicted to be highly suitable for *Bd* by all approaches. Similarly, a narrow region in northern Africa, ranging from Tunisia over Algeria to Morocco was predicted. ENMs for both regions were recently confirmed by respective positive *Bd* records [84], [85]. The causes for the differences between our ENMs and previous ones are complex. The models differ substantially in the parameter setting of the algorithm, their resolution, the points used, and the environmental parameters.

The origin of *Bd* is still unknown. One hypothesis was that the pathogen originated in Africa and spread globally via the commercial trade of clawed frogs (Pipidae: *Xenopus* spp.). Histological and molecular analyses [86], [87], detailed trade history [88] and known occurrences at that time [89], [90], [91] supported this hypothesis [see also 29], [92]. In addition the oldest known record originated from Cameroon, more specifically *Bd* was detected in a museum voucher of *Xenopus fraseri*, collected in 1933 from lowland rainforest [87]. Now an older record from Japan, dated to 1902 [93], challenges the hypothesis that *Bd* originated in Africa. However, there is more than one lineage of *Bd* [60], [93], [94] and one or more pathogenic lineages could have spread out of Africa.

This leads to the question of how *Bd* is transported from one location to another. Trade of live animals is commonly suggested as the most likely means of dispersal [4], [12], [64], [60], [65], [86], [94], [95], [96], [97], [98], [99]. However, recent findings support the notion that other dispersal vectors are also possible such as reptiles, birds or mammals [100], [101], [102].

### Potential Error Sources

We herein did not find any evidence for *Bd* in West Africa. Several explanations are plausible why *Bd* was not recorded in our study area. Either (i) sampling was flawed if *Bd* follows seasonal patterns and we sampled during a low prevalence cycle [e.g. 72], [103] or (ii) species were sampled whose ecological niches do not or only slightly overlap with that of *Bd* [e.g. 73], [104], [105]. Other possibilities are (iii) that sampling in the field failed, e.g. due to blemished preservation [e.g. 46], [106] or (iv) poor diagnostic assays, e.g. presence of PCR inhibitors [e.g. 107]. Although possible, it is unlikely that *Bd* was not detected due to aforementioned errors. Seasonality might be a problem. We sampled mainly during the wet season and even in highly seasonal *Bd* infected regions, positive confirmation is possible year round [108] though not everywhere [68], [73], [103], [109].

We sampled species with ecological requirements strongly overlapping with the fungus, including avoiding xeric species such as *Amietophrynus xeros* or *Tomopterna cryptotis*. Many of the sampled genera have previously been shown to be infected with *Bd* in other African regions (e.g.: *Amietophrynus* (mean prevalence 21.05%; Bayesian credible interval 11.13–36.46%), *Hyperolius* (39.51%; 35.26–43.92), *Leptopelis* (28.57%; 22.03–36.18%), *Petropedetes* (11.11%; 15.17–65.11%), *Phrynobatrachus* (17.65%; 9.63–30.32%), *Ptychadena* (26.26%; 20.36–33.17%), *Xenopus* (3.35%; 2.35–4.77%) [calculated from 26], [27], [28], [29], [85], [86], [90], [91], [92]).

Thirdly, anuran tissue samples, from which DNA was successfully extracted, were preserved following the same procedures as *Bd*-swabs and toe clips. In addition all methods used in this paper have already detected *Bd* in samples from other regions [see method section and 28], [86].

Lastly, amplification of DNA from frog 16 s and fungal ITS regions for the samples at UW (n = 103) demonstrate that swabbing was effective (see S2). In terms of numbers of individuals and geographical scale, our sample size is also large enough to make a confident diagnosis. All the above mentioned facts support our conclusion that our sampling is representative for West African amphibians and that *Bd* is highly likely to be absent in western Africa.

### Conservation implications

Though *Bd* has been detected in a number of species with different ecological niches, most populations which are adversely affected by the fungus are from higher altitudes and inhabit mostly flowing streams [see above and 105], [110]. Therefore three West African species are of major conservation concern with regards to *Bd* infection: *Nimbaphrynoides occidentalis* (samples tested herein: n = 62), *Conraua alleni* (n = 86) and *Petropedetes natator* (n = 158). The Nimba toad, *N. occidentalis*, is the only truly viviparous anuran species and is restricted to narrow ranges of high altitude grasslands of the Nimba Mountains, which are situated at the border between Guinea, Liberia and Côte d'Ivoire [111], [112 and citations therein]. This species is listed as “Critically Endangered” because of its very small distribution range and the decline of suitable habitats [113]. *C. alleni* and *P. natator* are frogs occurring in streams, mostly in mountainous forest habitats. They are listed as “Vulnerable” and “Near Threatened” respectively [113].

The geographic distributions of all three species show a high overlap with the potential geographic ENM distribution of *Bd*. The models highly predict the occurrence of *Bd* in areas where all three species can be found (Fig. 2d). The fact that *N. occidentalis* is independent of flowing streams does not necessarily render this species less susceptible to *Bd*, as *Bd* has already been detected in at least three African species without aquatic larval stages: *Nectophrynoides asperginis* [114], [115] (though note that the species lived (extinct in the wild) in the spray zone of Kihansi River Gorge, Tanzania), *Arthroleptis* sp. (in Gabon [29] and in Malawi [116]) and the suspected direct developer *Balebreviceps hillmani* (in Ethiopia [85]). *Bd* is also suspected to be responsible for the extinction of four other direct developing species ( = no aquatic larval stage): *Craugastor milesi* (from Honduras), *Rheobatrachus silus*, *R. vitellinus*, and *Taudactylus diurnus* (from Australia). Though heavy logging occurred in the areas of distribution of the Australian species as well and all four species are associated with water (*C. milesi* adults live along rivers; *R. silus* & *R. vitellinus* have aquatic adults; *T. diurnus* lays eggs in water) [1], [113]. Therefore, we conclude that *Bd* could potentially occur in western Africa due to the availability of suitable habitats and susceptible hosts.

Our sampling covers a representative subsample of West African species. This is not only due to the number of species sampled but also due to the fact that two species have been intensively sampled, which are habitat generalists (S1) and have a wide distribution, i.e. *Phrynobatrachus latifrons* (n = 79) and *Hoplobatrachus occipitalis* (n = 67) [117]. The latter species is also the major traded species in local and regional food markets and is therefore transported over long distances [118]. The species is also transported across the Dahomey Gap, more specifically from north-eastern Bénin to south-western Nigeria and probably even further eastwards [118], [119]. Thus the possibility that *Bd* will be spread from Nigeria to the west is reduced.

We will briefly highlight the most likely entry points for *Bd* from Central Africa to West Africa. Looking at the major transportation routes, a human *Bd* transport distribution will in all likelihood first be detected in the region around Accra (Fig. 2b). A highway exists parallel to the coast and connects the major cities (Lagos, Nigeria; Porto Novo & Cotonou in Bénin, Lomé, Togo; Accra, Ghana). Environmental suitability for *Bd* is low in Bénin and Togo, making Ghana a more likely entry point for *Bd*. Railways exist but mainly in north-south directions and rarely cross international borders. They operate also on a rare and infrequent basis and are not a major means of transportation. The introduction of *Bd* into West Africa via animate vectors is much more difficult to predict. The most likely entry point for them would be either the highlands of Togo or the Atewa range in Ghana (Fig. 2b), because they are closest to the *Bd* positive localities in Nigeria (Okomu NP) and are environmentally suitable for *Bd*.

Every effort has to be made to ensure that *Bd* will not invade western Africa, especially because threats are additive [e.g. 8] and fragmentation has already affected the region heavily [see 120], [121], [122]. The situation is similar to Madagascar where *Bd* has also not been detected [86], [123], [124]. For environmental work in the region (e.g. consultant, scientific) we strongly recommend buying new equipment. This has to include the disinfection of materials and equipment transported from *Bd* positive to *Bd* negative regions, especially to *Bd* sensitive regions for example by mining companies as these sensitive areas often coincide with proposed mining areas [see 125], [126], [127], [128], [129]. The same precautionary measures should apply for the ecotourism industry [see 130]. Only through acute scientific observation, greater collaboration between conservation and all sectors of industry and commerce can some measure of control be achieved over the spread of wildlife pathogens such as *Bd*.

## Supporting Information

Appendix S1 **List of West African caecilian and anuran species tested for the presence of** ***Bd*** **, and their main ecological characters.**
(PDF)

Appendix S2 **List of study areas, their geographic positions as well as details of sampling and analysis for each sample.**
(XLS)

Appendix S3 **List of positive African** ***Bd*** **records.**
(PDF)

Appendix S4 **Details of the variable contributions to the calculated ENMs.**
(PDF)
