# Supplementary material for: West Africa - A Safe Haven for Frogs? A Sub-Continental Assessment of the Chytrid Fungus (Batrachochytrium dendrobatidis)
Source: PLoS One. 2013 Feb 13;8(2):e56236. doi: 10.1371/journal.pone.0056236 (PMC3572032; doi:10.1371/journal.pone.0056236)
Supplement: Appendix S1 — List of West African caecilian and anuran species tested for the presence of Bd , and their main ecological characters. (PDF) [file pone.0056236.s001.pdf]

**S1. List of West African caecilian and anuran species tested for the presence of *Bd*, and their main ecological characters.** All tests provided negative results. * = taxa known to comprise complexes of cryptic species (Rödel *et al*. unpubl. data); § = comprises 52 samples from Guinea (*N. o. occidentalis*) and 10 samples from Liberia (*N. o. liberiensis*); # = unknown species sampled at the airport in Accra, Ghana; swab = molecular investigation of swab sample; toe/skin = histological examination of toe tip; lowland = most populations below 1,500 m asl; montane = most populations above 1,500 m asl; forest = predominantly undisturbed evergreen rainforest; farmbush = degraded forest, open areas in rainforest, dry forest; savannah = tree and open savannah, agricultural land; tadpoles = aquatic feeding larvae; direct dev = direct development without free larval stage, either in egg (*Arthroleptis* spp., *Phrynobatrachus tokba*) or female (*Nimbaphrynoides*); lentic = larval development in stagnant, usually temporary waters of varying sizes; lotic = larval development in flowing, usually permanent waters of varying sizes. Taxonomy based on most recent list of the "Amphibian species of the World" [1].

| | sample | | altitude | | habitat | | | reproductive mode | | breeding site | |
|---|---|---|---|---|---|---|---|---|---|---|---|
| | swab | toe/skin | lowland | montane | forest | farmbush | savannah | tadpoles | direct dev | lentic | lotic |
| **Gymnophiona** | | | | | | | | | | | |
| **Caecilidae** | | | | | | | | | | | |
| *Geotrypetes seraphini* | 0 | 6 | 1 | 0 | 1 | 1 | 0 | 0 | 1 | 0 | 0 |
| **Anura** | | | | | | | | | | | |
| **Arthroleptidae** | | | | | | | | | | | |
| *Arthroleptis* spp. * | 24 | 0 | 1 | 0 | 1 | 1 | 0 | 0 | 1 | 0 | 0 |
| *Astylosternus occidentalis* | 3 | 0 | 1 | 0 | 1 | 1 | 0 | 1 | 0 | 0 | 1 |
| *Cardioglossa occidentalis* | 2 | 0 | 1 | 0 | 1 | 0 | 0 | 1 | 0 | 0 | 1 |
| *Leptopelis spiritusnoctis* | 5 | 0 | 1 | 0 | 1 | 1 | 0 | 1 | 0 | 1 | 0 |
| *L. viridis* | 8 | 0 | 1 | 0 | 0 | 1 | 1 | 1 | 0 | 1 | 0 |
| **Bufonidae** | | | | | | | | | | | |
| *Amietophrynus maculatus* | 17 | 0 | 1 | 0 | 0 | 1 | 1 | 1 | 0 | 1 | 0 |
| *A. regularis* | 1 | 0 | 1 | 0 | 0 | 1 | 1 | 1 | 0 | 1 | 0 |
| *A. togoensis* | 3 | 0 | 1 | 0 | 1 | 0 | 0 | 1 | 0 | 0 | 1 |

| | | | | | | | | | | | |
|---|---|---|---|---|---|---|---|---|---|---|---|
| *“Bufo” pentoni* | 4 | 0 | 1 | 0 | 0 | 0 | 1 | 1 | 0 | 1 | 0 |
| *Nimbaphrynoides occidentalis* § | 62 | 0 | 0 | 1 | 0 | 0 | 1 | 0 | 1 | 0 | 0 |
| **Dicroglossidae** | | | | | | | | | | | |
| *Hoplobatrachus occipitalis* | 55 | 12 | 1 | 0 | 0 | 1 | 1 | 1 | 0 | 1 | 0 |
| **Hemisotidae** | | | | | | | | | | | |
| *Hemisus marmoratus* | 5 | 0 | 1 | 0 | 1 | 1 | 1 | 1 | 0 | 1 | 0 |
| **Hyperoliidae** | | | | | | | | | | | |
| *Afrixalus dorsalis* | 3 | 0 | 1 | 0 | 0 | 1 | 1 | 1 | 0 | 1 | 0 |
| *Afrixalus nigeriensis* | 1 | 0 | 1 | 0 | 1 | 1 | 0 | 1 | 0 | 1 | 0 |
| *Afrixalus vittiger* | 5 | 0 | 1 | 0 | 0 | 0 | 1 | 1 | 0 | 1 | 0 |
| *A. weidholzi* | 1 | 0 | 1 | 0 | 0 | 0 | 1 | 1 | 0 | 1 | 0 |
| *Hyperolius bobirensis* | 2 | 0 | 1 | 0 | 1 | 0 | 0 | 1 | 0 | 1 | 0 |
| *H. concolor* | 5 | 0 | 1 | 0 | 0 | 1 | 0 | 1 | 0 | 1 | 0 |
| *H. fusciventris* | 1 | 0 | 1 | 0 | 1 | 1 | 0 | 1 | 0 | 1 | 0 |
| *H. guttulatus* | 2 | 0 | 1 | 0 | 0 | 1 | 0 | 1 | 0 | 1 | 0 |
| *H. nasutus* * | 1 | 0 | 1 | 0 | 0 | 0 | 1 | 1 | 0 | 1 | 0 |
| *H. nitidulus* | 6 | 0 | 1 | 0 | 0 | 0 | 1 | 1 | 0 | 1 | 0 |
| *H. picturatus* | 2 | 0 | 1 | 0 | 0 | 1 | 0 | 1 | 0 | 1 | 0 |
| *H. sylvaticus* | 2 | 0 | 1 | 0 | 1 | 0 | 0 | 1 | 0 | 1 | 0 |
| *H.* sp. # | 9 | 0 | 1 | 0 | ? | ? | ? | 1 | 0 | 1 | 0 |
| *Kassina arboricola* | 2 | 0 | 1 | 0 | 1 | 1 | 0 | 1 | 0 | 1 | 0 |
| *K. cassinoides* | 2 | 0 | 1 | 0 | 0 | 0 | 1 | 1 | 0 | 1 | 0 |
| *K. schioetzi* | 3 | 0 | 1 | 0 | 0 | 1 | 1 | 0 | 0 | 1 | 0 |
| *K. senegalensis* | 12 | 0 | 1 | 0 | 0 | 0 | 1 | 1 | 0 | 1 | 0 |
| *Phlyctimantis boulengeri* | 2 | 0 | 1 | 0 | 1 | 1 | 0 | 1 | 0 | 1 | 0 |
| **Microhylidae** | | | | | | | | | | | |
| *Phrynomantis microps* | 33 | 0 | 1 | 0 | 0 | 0 | 1 | 1 | 0 | 1 | 0 |
| **Petropedetidae** | | | | | | | | | | | |
| *Conraua alleni* * | 52 | 34 | 1 | 1 | 1 | 0 | 0 | 1 | 0 | 0 | 1 |
| *C. derooi* | 5 | 7 | 1 | 0 | 1 | 0 | 0 | 1 | 0 | 0 | 1 |
| *Petropedetes natator* * | 138 | 20 | 1 | 1 | 1 | 0 | 0 | 1 | 0 | 0 | 1 |
| **Phrynobatrachidae** | | | | | | | | | | | |
| *Phrynobatrachus alleni* | 10 | 0 | 1 | 0 | 1 | 0 | 0 | 1 | 0 | 1 | 1 |

| | | | | | | | | | | | |
|---|---|---|---|---|---|---|---|---|---|---|---|
| *P. annulatus* | 9 | 0 | 1 | 0 | 1 | 0 | 0 | ? | ? | ? | ? |
| *P. calcaratus* * | 10 | 0 | 1 | 0 | 1 | 1 | 0 | 1 | 0 | 1 | 1 |
| *P. francisci* | 2 | 0 | 1 | 0 | 0 | 0 | 1 | 1 | 0 | 1 | 0 |
| *P. latifrons* | 43 | 36 | 1 | 0 | 0 | 1 | 1 | 1 | 0 | 1 | 0 |
| *P. liberiensis* | 19 | 0 | 1 | 0 | 1 | 0 | 0 | 1 | 0 | 0 | 1 |
| *P. natalenis* | 4 | 0 | 1 | 0 | 0 | 1 | 1 | 1 | 0 | 1 | 0 |
| *P. plicatus* | 19 | 0 | 1 | 0 | 1 | 0 | 0 | 1 | 0 | 1 | 0 |
| *P. tokba* | 2 | 0 | 1 | 1 | 1 | 1 | 0 | 0 | 1 | 0 | 0 |
| *P.* sp. | 1 | 0 | 1 | 0 | ? | ? | ? | ? | ? | ? | ? |
| **Pipidae** | | | | | | | | | | | |
| *Pseudhymenochirus merlini* | 0 | 1 | 1 | 0 | 1 | 1 | 0 | 1 | 0 | 1 | 0 |
| *Silurana tropicalis* | 6 | 9 | 1 | 0 | 1 | 1 | 0 | 1 | 0 | 1 | 0 |
| *Xenopus muelleri* | 2 | 5 | 1 | 0 | 0 | 0 | 1 | 1 | 0 | 1 | 0 |
| **Ptychadenidae** | | | | | | | | | | | |
| *Ptychadena aequiplicata* * | 5 | 0 | 1 | 0 | 1 | 0 | 0 | 1 | 0 | 1 | 0 |
| *P. bibroni* | 1 | 0 | 1 | 0 | 0 | 1 | 1 | 1 | 0 | 1 | 0 |
| *P. longirostris* | 5 | 0 | 1 | 0 | 1 | 0 | 0 | 1 | 0 | 1 | 0 |
| *P. mascareniensis* * | 1 | 0 | 1 | 0 | 0 | 1 | 1 | 1 | 0 | 0 | 1 |
| *P. oxyrhynchus* | 1 | 0 | 1 | 0 | 0 | 1 | 1 | 1 | 0 | 1 | 0 |
| *P. pumilio* | 5 | 0 | 1 | 0 | 0 | 0 | 1 | 1 | 0 | 1 | 0 |
| *P. tellini* | 4 | 0 | 1 | 0 | 0 | 0 | 1 | 1 | 0 | 1 | 0 |
| *P. tournieri* | 2 | 0 | 1 | 0 | 0 | 0 | 1 | 1 | 0 | 1 | 0 |
| **Pyxicephalidae** | | | | | | | | | | | |
| *Pyxicephalus edulis* | 5 | 0 | 1 | 0 | 0 | 0 | 1 | 1 | 0 | 1 | 0 |
| *Aubria subsigillata* | 2 | 7 | 1 | 0 | 1 | 0 | 0 | 1 | 0 | 1 | 1 |
| **Ranidae** | | | | | | | | | | | |
| *Hylarana albolabris* | 11 | 0 | 1 | 0 | 1 | 0 | 0 | 1 | 0 | 0 | 1 |
| *H. galamensis* | 6 | 0 | 1 | 0 | 0 | 0 | 0 | 1 | 0 | 1 | 0 |
| *H. occidentalis* | 2 | 0 | 1 | 0 | 1 | 0 | 0 | 1 | 0 | ? | ? |
| **Rhacophoridae** | | | | | | | | | | | |
| *Chiromantis rufescens* | 1 | 0 | 1 | 0 | 1 | 0 | 0 | 1 | 0 | 1 | 0 |

## References


1. Frost DR (2011) Amphibian Species of the World: an Online Reference. Version 5.5. http://research.amnh.org/vz/herpetology/amphibia/ Accessed 28 February 2012.
