# Supplementary material for: West Africa - A Safe Haven for Frogs? A Sub-Continental Assessment of the Chytrid Fungus (Batrachochytrium dendrobatidis)
Source: PLoS One. 2013 Feb 13;8(2):e56236. doi: 10.1371/journal.pone.0056236 (PMC3572032; doi:10.1371/journal.pone.0056236)
Supplement: Appendix S3 — List of positive African Bd records. (PDF) [file pone.0056236.s003.pdf]

S3. List of positive African *Bd* records. The list shows localities which were accurate on the 30 arc sec grid. Out of the first 177 points 112 points were used for the ENMs (the remaining 65 fell on the same grid cell as another positive record). Record numbers 178 to 180 show the old records which are depicted in the figures but not used for the ENMs. The following abbreviation was used: NP = National Park. Taxonomy adjusted to most recent list of the "Amphibian species of the World" [1] with original names in brackets.

No.	Country	Site	Lat	Lon	Genus	Species	Reference
1	Gabon	Monts de Cristal & Ivindo NP	0.51	12.80	*Afrixalus*	*paradorsalis*	[2]
2	Gabon	Monts de Cristal & Ivindo NP	0.63	10.40	*Hylarana* (*Amnirana*)	sp.	[2]
3	Gabon	Monts de Cristal & Ivindo NP	0.51	12.80	*Hylarana* (*Amnirana*)	sp.	[2]
4	Gabon	Monts de Cristal & Ivindo NP	0.52	12.79	*Hylarana* (*Amnirana*)	sp.	[2]
5	Gabon	Monts de Cristal & Ivindo NP	0.50	12.80	*Hylarana* (*Amnirana*)	sp.	[2]
6	Gabon	Monts de Cristal & Ivindo NP	0.51	12.80	*Arthroleptis*	sp.	[2]
7	Gabon	Monts de Cristal & Ivindo NP	0.45	10.28	*Arthroleptis*	sp.	[2]
8	Gabon	Monts de Cristal & Ivindo NP	0.45	10.28	*Cardioglossa*	*elegans*	[2]
9	Gabon	Monts de Cristal & Ivindo NP	0.45	10.28	*Chiromantis*	*rufescens*	[2]
10	Gabon	Monts de Cristal & Ivindo NP	0.51	12.80	*Chiromantis*	*rufescens*	[2]
11	Gabon	Monts de Cristal & Ivindo NP	0.51	12.80	*Chiromantis*	*rufescens*	[2]
12	Gabon	Monts de Cristal & Ivindo NP	0.50	12.79	*Conraua*	*crassipes*	[2]
13	Gabon	Monts de Cristal & Ivindo NP	0.45	10.28	*Hyperolius*	*ocellatus*	[2]
14	Gabon	Monts de Cristal & Ivindo NP	0.52	12.79	*Hyperolius*	*ocellatus*	[2]
15	Gabon	Monts de Cristal & Ivindo NP	0.50	12.79	*Hyperolius*	*ocellatus*	[2]
16	Gabon	Monts de Cristal & Ivindo NP	0.50	12.80	*Hyperolius*	*ocellatus*	[2]
17	Gabon	Monts de Cristal & Ivindo NP	0.62	10.41	*Hyperolius*	*tuberculatus*	[2]
18	Gabon	Monts de Cristal & Ivindo NP	0.50	12.79	*Leptopelis*	*aubryi*	[2]
19	Gabon	Monts de Cristal & Ivindo NP	0.50	12.79	*Leptopelis*	*aubryi*	[2]
20	Gabon	Monts de Cristal & Ivindo NP	0.50	12.80	*Leptopelis*	*aubryi*	[2]
21	Gabon	Monts de Cristal & Ivindo NP	0.51	12.80	*Leptopelis*	*brevirostris*	[2]
22	Gabon	Monts de Cristal & Ivindo NP	0.63	10.40	*Leptopelis*	*crystallinoron*	[2]
23	Gabon	Monts de Cristal & Ivindo NP	0.45	10.28	*Leptopelis*	*crystallinoron*	[2]
24	Gabon	Monts de Cristal & Ivindo NP	0.51	12.80	*Leptopelis*	*millsoni*	[2]
25	Gabon	Monts de Cristal & Ivindo NP	0.62	10.40	*Leptopelis*	sp.	[2]

26	Gabon	Monts de Cristal & Ivindo NP	0.51	12.80	*Leptopelis*	sp.	[2]
27	Gabon	Monts de Cristal & Ivindo NP	0.50	12.80	*Leptopelis*	sp.	[2]
28	Gabon	Monts de Cristal & Ivindo NP	0.63	10.40	*Petropedetes*	*vulpiae* (*newtoni*)	[2]
29	Gabon	Monts de Cristal & Ivindo NP	0.45	10.28	*Petropedetes*	*palmipes*	[2]
30	Gabon	Monts de Cristal & Ivindo NP	0.45	10.28	*Petropedetes*	*palmipes*	[2]
31	Gabon	Monts de Cristal & Ivindo NP	0.45	10.28	*Phrynobatrachus*	*auritus*	[2]
32	Gabon	Monts de Cristal & Ivindo NP	0.63	10.40	*Phrynobatrachus*	*auritus*	[2]
33	Gabon	Monts de Cristal & Ivindo NP	0.63	10.40	*Phrynobatrachus*	*auritus*	[2]
34	Gabon	Monts de Cristal & Ivindo NP	0.45	10.28	*Ptychadena*	sp.	[2]
35	Gabon	Monts de Cristal & Ivindo NP	0.50	12.79	*Ptychadena*	sp.	[2]
36	Gabon	Monts de Cristal & Ivindo NP	0.45	10.28	*Scotobleps*	*gabonicus*	[2]
37	Gabon	Monts de Cristal & Ivindo NP	0.63	10.40	*Scotobleps*	*gabonicus*	[2]
38	Gabon	Monts de Cristal & Ivindo NP	0.62	10.41	*Scotobleps*	*gabonicus*	[2]
39	Gabon	Monts de Cristal & Ivindo NP	0.51	12.80	*Afrixalus*	*fulvovittatus*	[2]
40	Kenya	Langata, Nairobi	-1.40	36.77	*Ptychadena*	*anchietae*	[3]
41	Malawi	Mulanje	-16.02	35.50	*Afrixalus*	*aureus crotalus*	[4]
42	Malawi	Mulanje	-16.02	35.52	*Amietia*	*angolensis*	[4]
43	Malawi	Mulanje	-15.90	35.67	*Amietia*	*angolensis*	[4]
44	Malawi	Mulanje	-15.96	35.69	*Amietia*	*angolensis*	[4]
45	Malawi	Mulanje	-15.96	35.69	*Amietia*	*angolensis*	[4]
46	Malawi	Mulanje	-15.96	35.69	*Amietia*	*johnstoni*	[4]
47	Malawi	Mulanje	-15.96	35.69	*Amietia*	*johnstoni*	[4]
48	Malawi	Mulanje	-15.82	35.72	*Arthroleptis*	*xenodactyloides*	[4]
49	Malawi	Mulanje	-15.82	35.72	*Arthroleptis*	*xenodactyloides*	[4]
50	Malawi	Mulanje	-15.89	35.62	*Nothophryne*	*broadleyi*	[4]
51	Malawi	Mulanje	-16.02	35.52	*Phrynobatrachus*	*natalensis*	[4]
52	Malawi	Mulanje	-15.82	35.72	*Phrynobatrachus*	*natalensis*	[4]
53	Malawi	Mulanje	-15.93	35.68	*Strongylopus*	*fuelleborni*	[4]
54	Malawi	Mulanje	-16.02	35.50	*Xenopus*	*muelleri*	[4]
55	Morocco	Agnane, Near Tetouan	35.54	-5.39	*Discoglossus*	*scovazzi*	[5]
56	Morocco	20km from Larache-Lakslakbir	35.04	-6.05	*Discoglossus*	*scovazzi*	[5]
57	Morocco	20km from Larache-Lakslakbir	35.04	-6.05	*Hyla*	*meridionalis*	[5]
58	Morocco	Larache	35.04	-6.03	*Pelobates*	*varavaldii*	[5]
59	DR Congo	Kahuzi Biega NP	-2.31	28.76	*Hyperolius*	*kivuensis*	[6]

60	DR Congo	Kahuzi Biega NP	-1.89	28.45	*Hyperolius*	*kuligae*	[6]
61	South Africa	Algeria, Cederberg	-32.37	19.06	*Amietia* (*Afrana*)	*fuscigula*	[7]
62	South Africa	Bloukikerboom-water, Geogap	-29.63	18.01	*Amietia* (*Afrana*)	*fuscigula*	[7]
63	South Africa	Grobbelaars River	-33.42	22.24	*Amietia* (*Afrana*)	*fuscigula*	[7]
64	South Africa	Groot Winterhoek	-33.00	19.06	*Amietia* (*Afrana*)	*fuscigula*	[7]
65	South Africa	Jamaka farm pond. Cederberg	-32.34	19.02	*Amietia* (*Afrana*)	*fuscigula*	[7]
66	South Africa	Kraaifontein, Geogap	-29.63	18.03	*Amietia* (*Afrana*)	*fuscigula*	[7]
67	South Africa	Stellenbosch	-33.93	18.87	*Amietia* (*Afrana*)	*fuscigula*	[7]
68	South Africa	Swellendam	-34.01	20.46	*Amietia* (*Afrana*)	*fuscigula*	[7]
69	South Africa	Table Mountain	-33.95	18.43	*Amietia* (*Afrana*)	*fuscigula*	[7]
70	South Africa	Table Mountain	-33.95	18.43	*Amietia* (*Afrana*)	*fuscigula*	[7]
71	South Africa	Tradouws pass	-33.97	20.70	*Amietia* (*Afrana*)	*fuscigula*	[7]
72	South Africa	Jamaka farm pond. Cederberg	-32.34	19.02	*Strongylopus*	*grayii*	[7]
73	Lesotho	Katsi Dam	-29.34	28.51	*Amietia* (*Afrana*)	*dracomontana*	[8]
74	South Africa	Kammieskroon, Northern Cape	-30.2	17.93	*Amietia* (*Afrana*)	*fuscigula*	[8]
75	South Africa	Springbok, Northern Cape	-26.85	21.78	*Amietia* (*Afrana*)	*fuscigula*	[8]
76	Lesotho	Katsi Dam	-29.34	28.51	*Amietia*	*vertebralis*	[8]
77	South Africa	Springbok, Northern Cape	-26.85	21.78	*Vandijkophrynus* (*Bufo*)	*robinsoni*	[8]
78	South Africa	Kenton on Sea	-33.70	26.68	*Cacosternum*	*boettgeri*	[8]
79	South Africa	Oudtshoorn	-33.58	22.20	*Heleophryne*	*regis*	[8]
80	South Africa	Kenton on Sea	-33.70	26.68	*Kassina*	*senegalensis*	[8]
81	South Africa	Kenton on Sea	-33.70	26.68	*Strongylopus*	*fasciatus*	[8]
82	South Africa	Zeekoevlei, Western Cape	-34.07	18.52	*Xenopus*	*gilli*	[8]
83	Botswana	Kanye Youth Centre	-24.98	25.35	*Xenopus*	*laevis*	[8]
84	South Africa	Florisbad, Free State	-28.77	26.08	*Xenopus*	*laevis*	[8]
85	South Africa	Harrismith, Free State	-28.28	29.13	*Xenopus*	*laevis*	[8]
86	South Africa	Klapmuts	-33.81	18.86	*Xenopus*	*laevis*	[8]
87	South Africa	Koffiefontein	-29.40	25.02	*Xenopus*	*laevis*	[8]
88	South Africa	Kwa-Zulu, Natal	-30.98	29.23	*Xenopus*	*laevis*	[8]
89	South Africa	Sannaspos	-29.15	26.53	*Xenopus*	*laevis*	[8]
90	South Africa	Strand	-34.12	18.83	*Xenopus*	*laevis*	[8]
91	South Africa	Zeekoevlei, Western Cape	-34.07	18.52	*Xenopus*	*laevis*	[8]
92	Zambia	Lusaka	-15.02	28.23	*Xenopus*	*laevis*	[8]
93	Zambia	Lusaka	-15.02	28.23	*Xenopus*	*laevis*	[8]

94	Nigeria	Okomu National Park	6.30	5.25	*Chiromatis*	*rufescens*	[9]
95	Kenya	Mt. Elgon NP	1.03	34.77	NA	NA	[10]
96	Kenya	Saiwa Swamp NP	1.11	35.12	NA	NA	[10]
97	Kenya	Shimba Hills NP	-4.18	39.42	NA	NA	[10]
98	Kenya	Taita Hills (Mwatate)	-3.51	38.38	NA	NA	[10]
99	Kenya	Taita Hills (Mwundanyi)	-3.41	38.36	NA	NA	[10]
100	Kenya	Aberdares NP (moorlands)	-0.41	36.72	NA	NA	[10]
101	Kenya	Aberdares NP (Salient)	-0.37	36.84	NA	NA	[10]
102	Kenya	Kakamega Forest NP	0.35	34.87	NA	NA	[10]
103	Kenya	Nairobi (Karens)	-1.33	36.80	NA	NA	[10]
104	Kenya	Thompson Falls	0.04	36.37	NA	NA	[10]
105	Kenya	Tigoni Dam	-1.14	36.68	NA	NA	[10]
106	South Africa	Stutterheim, Kologha Forest	-32.54	27.37	*Amietia* (*Afrana*)	*fuscigula*	[11]
107	South Africa	Grabouw	-34.15	19.02	*Xenopus*	*laevis*	[12]
108	South Africa	Bredasor/Bredasdorp?	-34.53	20.03	*Xenopus*	*laevis*	[12]
109	South Africa	Hex River	-33.48	19.58	*Xenopus*	*laevis*	[12]
110	South Africa	Knysna	-34.03	23.03	*Xenopus*	*laevis*	[12]
111	Nigeria	Kwano Camp (Gashaka Gumti NP)	7.33	11.59	*Amietophrynus*	sp.	[13]
112	Nigeria	Kwano Camp (Gashaka Gumti NP)	7.33	11.59	*Astylosternus*	sp.	[13]
113	Nigeria	Kwano Camp (Gashaka Gumti NP)	7.33	11.59	*Petropedetes*	sp.	[13]
114	Tanzania	Kihansi falls	-8.59	35.85	*Nectophrynoides*	*asperginis*	[14]
115	South Africa	Bela-Bela, Limpopo Province	-24.88	28.29	*Amietia* (*Afrana*)	*angolensis*	[15]
116	South Africa	Port Elizabeth, Eastern Cape	-33.97	25.58	*Amietia* (*Afrana*)	*fuscigula*	[15]
117	South Africa	Bela-Bela, Limpopo Province	-24.88	28.29	*Tomopterna*	*cryptotis*	[15]
118	South Africa	Bela-Bela, Limpopo Province	-24.88	28.29	*Tomopterna*	*natalensis*	[15]
119	South Africa	Botrivier, Western Cape	-34.23	19.20	*Xenopus*	*laevis*	[15]
120	South Africa	Wellington, Western Cape	-33.63	19.00	*Xenopus*	*laevis*	[15]
121	South Africa	Kommissiepoort, Free State	-29.32	27.28	*Xenopus*	*laevis*	[16]
122	South Africa	Moi River, KwaZulu-Natal	-29.20	29.98	*Xenopus*	*laevis*	[16]
123	South Africa	Phillipi, Western Cape	-34.02	18.55	*Xenopus*	*laevis*	[16]
124	South Africa	Rosendal, Free State	-28.52	27.93	*Xenopus*	*laevis*	[16]
125	South Africa	Windsorton Road, Northern Cape	-28.35	24.82	*Xenopus*	*laevis*	[16]
126	Cameroon	Ngoum-Bandi (PK27)	2.14	15.66	*Phlyctimantis*	*boulengeri*	[17]
127	Cameroon	Mt Oku	6.22	10.39	NA	NA	[18]

128	Cameroon	Bangabakundu	4.41	9.45	NA	NA	[18]
129	Cameroon	Mt Oku	6.25	10.52	NA	NA	[18]
130	Cameroon	Mt Oku	6.19	10.46	NA	NA	[18]
131	Cameroon	Mt Oku	6.19	10.46	NA	NA	[18]
132	Cameroon	Mt Cameroon	4.18	9.20	NA	NA	[18]
133	Cameroon	Mt Oku	6.24	10.52	NA	NA	[18]
134	Cameroon	Mt Oku	6.21	10.46	NA	NA	[18]
135	Cameroon	Mundame	4.56	9.52	NA	NA	[18]
136	Cameroon	Ndikinimei	4.75	10.82	NA	NA	[18]
137	Cameroon	Ndikinimei	4.75	10.83	NA	NA	[18]
138	Cameroon	Ndikinimei	4.76	10.81	NA	NA	[18]
139	Cameroon	Mt Oku	6.20	10.46	NA	NA	[18]
140	Cameroon	Ntengue	5.37	10.02	NA	NA	[18]
141	Cameroon	Manengouba village	4.86	9.86	*Cardioglossa*	*melanogaster*	[18]
142	Cameroon	Manengouba village	4.96	9.87	*Leptodactylodon*	*mertensi*	[18]
143	Cameroon	Manengouba village	4.96	9.87	*Leptopelis*	*calcaratus*	[18]
144	Cameroon	Manengouba village	4.95	9.88	*Phrynobatrachus*	*africanus*	[18]
145	Cameroon	Mt Manengouba	5.07	9.87	*Cardioglossa*	*gracilis*	[18]
146	Cameroon	Mt Manengouba	5.07	9.87	*Cardioglossa*	*gracilis*	[18]
147	Cameroon	Mt Manengouba	5.07	9.87	*Cardioglossa*	*gracilis*	[18]
148	Cameroon	Mt Manengouba	5.07	9.87	*Cardioglossa*	*gracilis*	[18]
149	Cameroon	Mt Manengouba	5.07	9.87	*Cardioglossa*	*melanogaster*	[18]
150	Cameroon	Mt Manengouba	5.04	9.86	*Arthroleptis*	*perreti*	[18]
151	Cameroon	Mt Manengouba	5.04	9.86	*Leptodactylodon*	*erythrogaster*	[18]
152	Cameroon	Ebonemin	5.01	9.77	*Leptopelis*	*brevirostris*	[18]
153	Cameroon	Ebonemin	5.01	9.77	*Chlorolius*	*koehleri*	[18]
154	Cameroon	Ebonemin	5.01	9.78	*Leptodactylodon*	*mertensi*	[18]
155	Cameroon	Ebonemin	5.02	9.76	*Leptopelis*	*calcaratus*	[18]
156	Cameroon	Nkack	5.04	9.77	*Arthroleptis*	*tuberosus*	[18]
157	Cameroon	Nkack	5.04	9.77	*Phrynobatrachus*	*cricogaster*	[18]
158	Cameroon	Mt Manengouba	5.01	9.82	*Leptodactylodon*	*erythrogaster*	[18]
159	Cameroon	Mt Manengouba	5.01	9.82	*Leptopelis*	*modestus*	[18]
160	Cameroon	Mt Manengouba	5.01	9.82	*Arthroleptis*	*perreti*	[18]
161	Cameroon	Mt Manengouba	5.01	9.82	*Arthroleptis*	*perreti*	[18]

162	Cameroon	Mt Manengouba	5.04	9.81	*Arthroleptis*	*perreti*	[18]
163	Cameroon	Mt Manengouba	5.03	9.81	*Leptodactylodon*	*erythrogaster*	[18]
164	Cameroon	Ebo Forest Reserve	4.35	10.23	*Cardioglossa*	*leucomystax*	[18]
165	Ethiopoa	Shawe bridge	6.65	39.73	NA	NA	[19]
166	Ethiopoa	Katcha	6.72	39.73	NA	NA	[19]
167	Ethiopoa	Rira	6.76	39.73	NA	NA	[19]
168	Ethiopoa	Fute	6.76	39.75	NA	NA	[19]
169	Ethiopoa	Tulla Negresso	6.78	39.75	NA	NA	[19]
170	Ethiopoa	Bonga town	7.27	36.26	NA	NA	[19]
171	Ethiopoa	Bonga stream	7.27	36.26	NA	NA	[19]
172	Ethiopoa	Bonga marsh	7.25	36.26	NA	NA	[19]
173	Ethiopoa	Mankira	7.20	36.29	NA	NA	[19]
174	Ethiopoa	Koma forest	7.32	36.08	NA	NA	[19]
175	Ethiopoa	Koma marsh	7.31	36.08	NA	NA	[19]
176	Ethiopoa	Wush wush	7.31	36.12	NA	NA	[19]
177	Ethiopoa	Saja forest	7.49	36.09	NA	NA	[19]
178	Cameroon	Batouri District	4.43	14.34	*Xenopus*	*fraseri*	[20]
179	Uganda	Lake Bunyoni	-1.23	29.82	*Xenopus*	*laevis bunyoniensis*	[20]
180	Malawi	Lilongwe	-13.98	33.78	*Xenopus*	*laevis laevis*	[20]

References

1. Frost DR (2011) Amphibian Species of the World: an Online Reference. Version 5.5. http://research.amnh.org/vz/herpetology/amphibia/ Accessed 28 February 2012.

2. Bell RC, Garcia AVG, Stuart BL, Zamudio KR (2011) High prelevance of the amphibian chytrid pathogen in Gabon. EcoHealth 8: 116-120.

3. Berger L, Parker JM (1999) In: Speare R, Berger L. (2000) Global distribution of chytridiomycosis in amphibians. Available: http://www.jcu.edu.au/school/phtm/PHTM/frogs/chyglob.htm. Accessed: 26 April 2011.

4. Conradie W, Harvey J, Kotze A, Dalton DL, Cunningham MJ (2011) Confirmed amphibian chytrid in Mount Mulanje Area, Malawi. Herpetol Rev 42: 369-371.

5. El Mouden EH, Slimani T, Donaire D, Fernández-Beaskoetxea S, Fisher MC, et al. (2011) First Record of the chytrid fungus *Batrachochytrium dendrobatidis* in North Africa. Herpetol Rev 42: 71-75.

6. Greenbaum E, Kusamba C, Aristote MM, Reed K (2008) Amphibian chytrid fungus infections in *Hyperolius* (Anura: Hyperoliidae) from eastern Democratic Republic of Congo. Herpetol Rev 39: 70-73.
7. Hopkins S, Channing A (2003) Chytrid fungus in northern and western Cape frog populations, South Africa. Herpetol Rev 34: 334-336.
8. Aanensen DM (2011) *Bd*-Maps. www.bd-maps.net. Accessed 26 April 2011.
9. Imasuen AA, Aisen MSO, Weldon C, Dalton DL, Kotze A, et al. (2011) Occurrence of *Batrachochytrium dendrobatidis* in Amphibian Populations of Okomu National Park, Nigeria. Herpetol Rev 42: 379-382.
10. Kielgast J, Rödder D, Veith M, Lötters S (2010) Widespread occurrence of the amphibian chytrid fungus in Kenya. Anim Conserv 13: 1-8.
11. Lane E, Weldon C, Bingham J (2003) Histological evidence of chytridiomycosis in a free-ranging amphibian (*Afrana fuscigula* (Anura: Ranidae)) in South Africa. J S Afr Vet Assoc 74: 20-21.
12. Mendez D, Speare R, Cunningham AA (1999) In: Speare R, Berger L. (2000) Global distribution of chytridiomycosis in amphibians. Available: http://www.jcu.edu.au/school/phtm/PHTM/frogs/chyglob.htm. Accessed: 26 April 2011.
13. Reeder NMM, Cheng TL, Vredenburg VT, Blackburn DC (2011) Survey of the chytrid fungus *Batrachochytrium dendrobatidis* from montane and lowland frogs in eastern Nigeria. Herpetol Notes 4: 83-86.
14. Weldon C, de Villiers L, du Preez LH (2007) Quantification of the trade in *Xenopus laevis* from South Africa, with implications for biodiversity conservation. Afr J Herpetol 56: 77-83.
15. Weldon C (2005) Chytridiomycosis, an emerging infectious disease of amphibians in South Africa. PhD Thesis. Potchefstroom, North-West University. 213 p.
16. Weldon C, du Preez LH, Hyatt AD, Muller R, Speare R. (2004) Origin of the amphibian chytrid fungus. Emerg Infect Diseases 10: 2100-2105.
17. Baláž V, Kopecký O, Gvoždík V (2012) Presence of the amphibian chytrid pathogen confirmed in Cameroon. Herp. J. 22: 191-194.
18. Doherty-Bone TM, Gonwouo NL, Hirschfeld M, Ohst T, Weldon C et al. (in press) *Batrachochytrium dendrobatidis* in amphibians of Cameroon, including first records of infected caecilian hosts. Dis Aquat Org.
19. Gower DJ, Doherty-Bone TM, Aberra RK, Mengistu A, Schwaller S et al. 2012) High prevalence of the amphibian chytrid fungus (*Batrachochytrium dendrobatidis*) across multiple taxa and localities in the highlands of Ehtiopia. Herp. J. 22: 225-233.
20. Soto-Azat C, Clarke BT, Poynton JC, Cunningham AA (2010) Widespread historical presence of *Batrachochytrium dendrobatidis* in African pipid frogs. Divers Distrib 16: 126-131.
