# Supplementary material for: West Africa - A Safe Haven for Frogs? A Sub-Continental Assessment of the Chytrid Fungus (Batrachochytrium dendrobatidis)
Source: PLoS One. 2013 Feb 13;8(2):e56236. doi: 10.1371/journal.pone.0056236 (PMC3572032; doi:10.1371/journal.pone.0056236)

S4. Details of the variable contributions to the calculated ENMs. Figures S4.1 to S 4.17 show the mean individual response curves (red) and their standard deviation (blue). Figure S4.18 details the estimated relative variable contribution without (percentage contribution) and with random permutation (permutation importance) of the values. Figure S4.19 and S4.20 show the results of the jackknifing tests of variable importance for the training (S4.19) and testing data (S4.20).

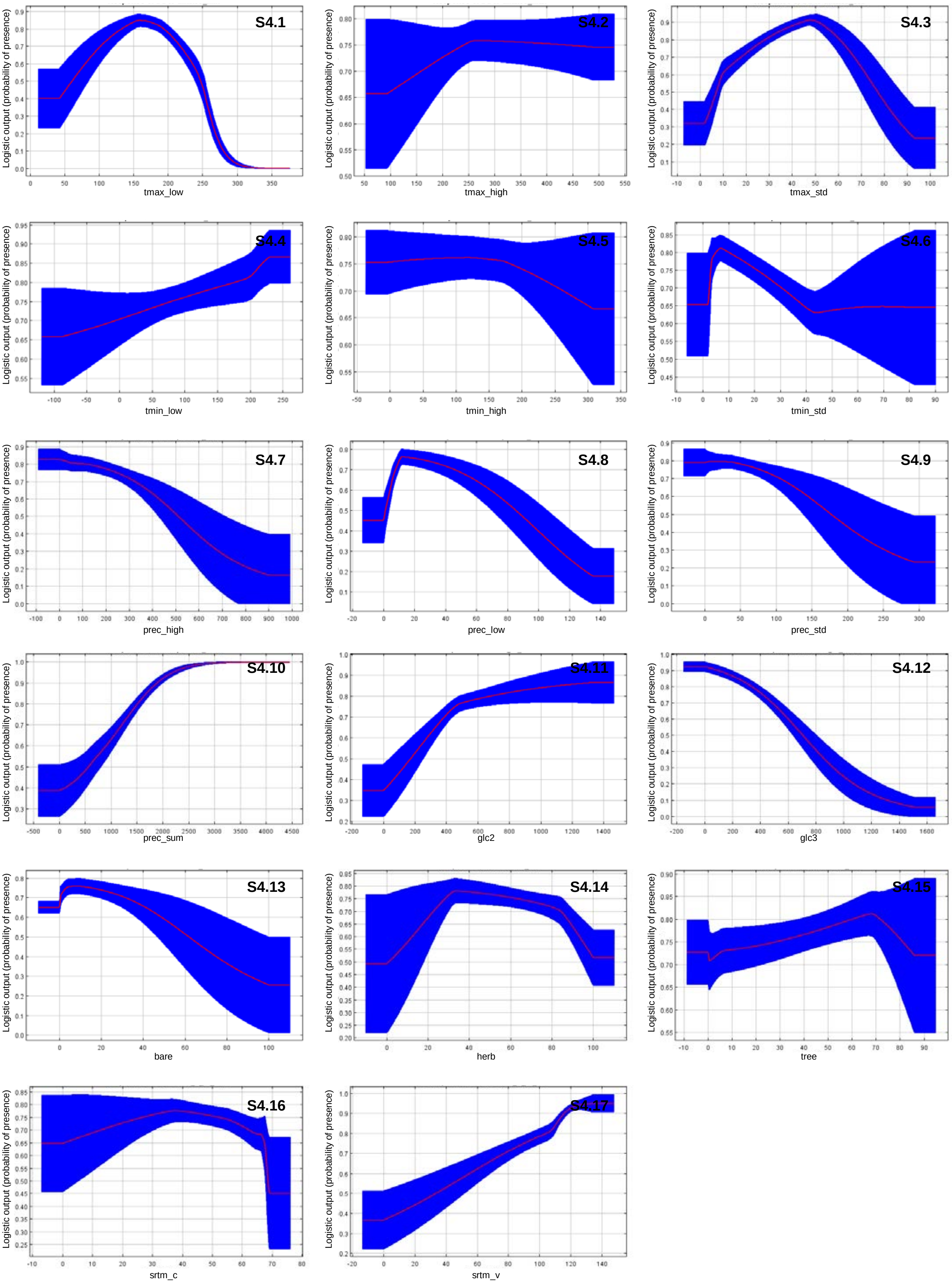

S4.18	Parameter	Percent contribution	Permutation importance
1	tmax_low	17.5	41.3
2	tmax_high	1.1	0.0
3	tmax_std	3.0	4.6
4	tmin_low	0.2	0.6
5	tmin_high	0.9	0.1
6	tmin_std	0.6	1.3
7	prec_high	0.1	1.2
8	prec_low	35.3	2.2
9	prec_std	0.2	1.0
10	prec_sum	5.0	12.3
11	glc2	0.5	1.5
12	glc3	1.3	11.8
13	bare	4.2	13.4
14	herb	3.2	3.9
15	tree	1.8	1.0
16	srtm_c	2.4	0.3
17	srtm_v	22.6	3.4

S4.19

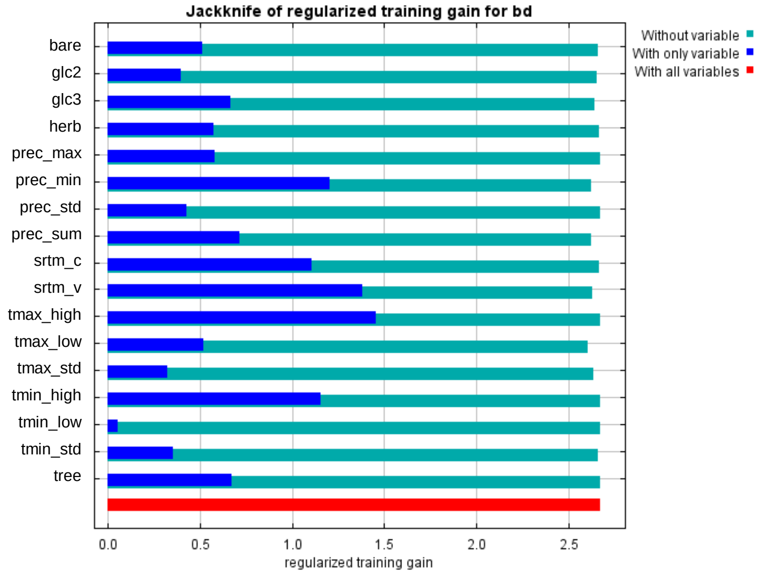

S4.20

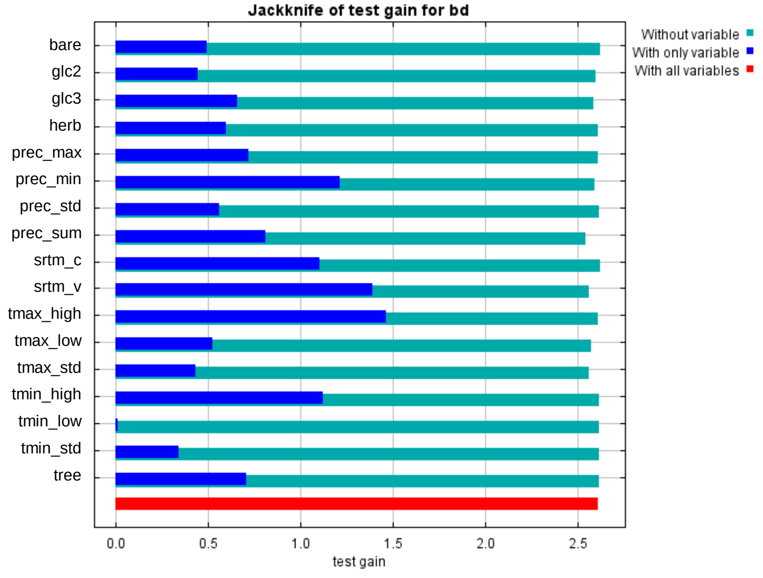

Supplement: Appendix S4 — Details of the variable contributions to the calculated ENMs. (PDF) [file pone.0056236.s004.pdf]
